# The Role of Probiotics in Chronic Rhinosinusitis Treatment: An Update of the Current Literature

**DOI:** 10.3390/healthcare9121715

**Published:** 2021-12-12

**Authors:** Maria Rita Bianco, Massimo Ralli, Domenico Michele Modica, Marta Amata, Salvatore Poma, Gianfranco Mattina, Eugenia Allegra

**Affiliations:** 1 Otolaryngology-Department of Health Science, University of Catanzaro, 88100 Catanzaro, Italy; eualle@unicz.it; 2 Department of Sense Organs, Sapienza University of Rome, 00185 Rome, Italy; massimo.ralli@uniroma1.it; 3 Otolaryngology Unit-“Villa Sofia”-Cervello Hospital, 90146 Palermo, Italy; domenicomichelemodica@gmail.com (D.M.M.); s.poma@alica.it (S.P.); mattina.gianfranco@gmail.com (G.M.); 4 Department of Biomedicine and Internal and Specialistic Medicine (DIBIMIS), University of Palermo, 90133 Palermo, Italy; marta.amata@gmail.com

**Keywords:** chronic rhinosinusitis, probiotics, microbiome, nasal microbiota, microbiome therapy

## Abstract

Chronic rhinosinusitis (CRS) is a significant health problem. It affects 5–12% of the general population. The causes that underlie the onset of CRS are not yet well known. However, many factors may contribute to its onset, such as environmental factors and the host’s general condition. Medical treatment mainly uses local corticosteroids, nasal irrigation, and antibiotics. In recent years, a new therapeutic approach that employs the use of probiotics emerged. Probiotics have been extensively studied as a therapy for dysbiosis and inflammatory pathologies of various parts of the body. We aimed to examine the studies in vivo and in vitro and clinicals reports in the existing literature to update probiotics’ role in rhinosinusitis chronic medical treatment.

## 1. Introduction

Chronic rhinosinusitis (CRS) is a significant health problem affecting 5–12% of the general population [1]. Furthermore, it is referred to as chronic when the inflammatory process persists for more than 12 consecutive weeks [2]. The investigation conducted by the Global Allergy and Asthma European Network (GALEN) in 2011 concluded that the prevalence of CRS in Europe amounts to 10.9%—between 6.9% and 27.1% in different European cities [3]. This pathology negatively impacts patients’ quality of life. Therefore, it must be correctly identified and treated.

The causes that underlie the onset of CRS are not yet well known. However, many factors may contribute to its onset, such as environmental factors (temperature, humidity, and air pollution) and the host’s general condition (anatomical variants, allergies, local or systemic immune system imbalance, and genetic predisposition) [4,5]. Medical treatment mainly uses local corticosteroids, nasal irrigation, and antibiotics; however, there is a scarcity of information in the literature about the use of nasal spray medical formulations for local treatment of CRS [6]. If medical treatment is insufficient, endoscopic sinus surgery is proposed. In recent years, a new therapeutic approach that employs the use of probiotics has emerged [7]. The World Health Organization (WHO) defines probiotics as products containing live microorganisms that, when administered in the right amounts, have a beneficial effect on the host’s health [8]. Probiotics have been extensively studied as a therapy for dysbiosis (imbalance of the microbial flora of a given habitat, or alteration of its composition or function) and inflammatory pathologies of various parts of the body (the digestive system and upper and lower airways) [9,10,11,12,13]. Studies conducted to evaluate the effect of probiotics on CRS are still few in number. Furthermore, there are concerns regarding experimental studies on animal models, including in vitro and clinical studies. We aimed to examine the studies in the existing literature to update the role of probiotics in rhinosinusitis chronic medical treatment.

## 2. Microbiota

A microbiota is described as a community of microorganisms that resides in a distinct environment, and the collection of entire genomic elements of a distinct microbiota is the microbiome [14]. In the various microenvironments of our organism, 10–100 trillion colonies of microorganisms coexist synergistically, constituting a commensal microbiome capable of coexisting within our organism and defending it from insults coming from the external environment. Since childhood, the microbiota composition tends to change to adapt to various internal and external stimuli during the normal physiological growth of tissues [15]. Humans acquire significant quantities of microbiota from the mother during birth by natural means [16]. This microbiota changes within the first three years of life and then becomes more stable, but is always subject to minor changes in the later stages of life [17].

Each site of the organism is colonized by various microorganisms (microbiota), characterizing the inhabited site, which is not constant but variable according to the subject’s age. With their complex and tortuous anatomy, the nasal cavities define anatomical spaces where we find different microbiota. Consequently, in the nasal vestibule—the nasal cavities and the paranasal sinuses—there exist diverse microbiota residents.

**Figure 1:**
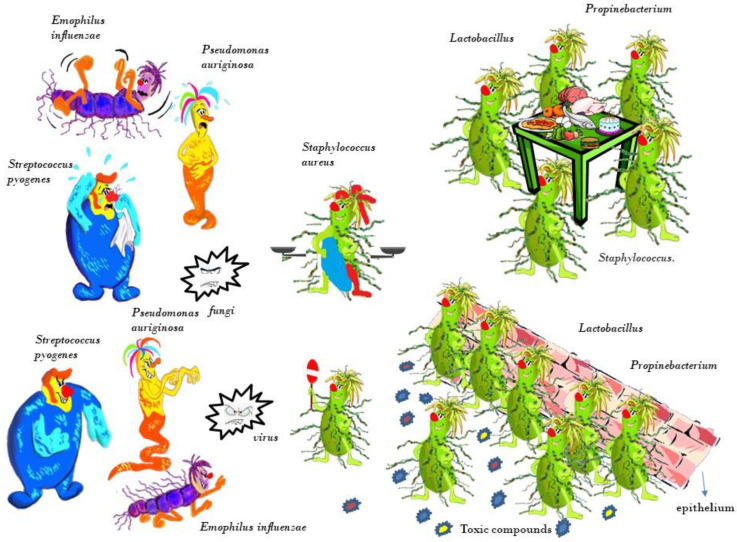
Resident commensal bacteria are involved in the homeostasis of the nasal microbiota, controlling and suppressing opportunistic pathogens, through a competition mechanism for spaces and nutrients. Additionally, products with toxic compounds inhibit or directly kill competing microorganisms (lactic acid, antibacterial peptides, and hydrogen peroxide). Opportunistic bacteria (see *Staphylococcus aureus*) retain the ability to act as commensals or pathogens.

Resident commensal bacteria are involved in the homeostasis of the nasal microbiota, controlling and suppressing opportunistic pathogens, through a competition mechanism for spaces and nutrients. Additionally, products with toxic compounds inhibit or directly kill competing microorganisms (lactic acid, antibacterial peptides, and hydrogen peroxide; Figure 1). 

The commensal bacteria are also an essential part of the nasal flora, and contribute to maintaining this district’s physiological and immune functions. Several studies show that failure balancing and dysbiosis [18] between the microorganisms (due to the use of antimicrobials, lifestyle factors, and factors dietary) determine an increase in opportunistic pathogens, favoring an alteration of the immune functions and increased infectious processes. The dysmicrobism of the upper respiratory tract can cause chronic inflammation of the airways, resulting in the spread of microorganisms in the various sections of the airways, thus generating pathological conditions, such as rhinosinusitis, chronic otitis media, asthma, and the worsening of the course of allergic rhinitis [19,20].

## 3. Nasal Microbiota

Recent studies have shown that microorganisms that make up the microbiota of the nasopharynx are different to those present in paranasal sinuses. Therefore, it is evident that there are several varieties of microbiota in the various sections of the upper respiratory tract [15].

As evidenced by Yan M. et al. [21], the nasal cavities constitute a transition zone between the external environment exposed to constant insults and the protected internal environment. The nose is an environment deficient in nutrients which is acidic and salty. The higher the acidity and salinity (nostrils), the greater the difficulty of microbial colonization. The nasal mucus, also containing small amounts of nutrients, restricts microbial growth, and, as such, only particular species and microbes have adapted to this environment. In nasal microbiota, phyla Actinobacteria *(Corynebacterium and Cutibacterium),* Firmicutes *(Staphylococcus, Streptococcus, Dolosigranulum, and Lactobacillus),* and Proteobacteria *(Moraxella and Haemophilus*), whose abundance varies depending on the portion of the nose, are commonly identified (Table 1). Among the phyla present in the nasal cavities, Actinobacteria are the predominant phyla, and are present at all stages of life [22]. Furthermore, they display patterns of microorganisms that tend to change over time. The growth of the individual (typically Proteobacteria) and more stable patterns (*Staphylococcus* epidermidis of the species Firmicutes) tend to be maintained over time.

The commensal bacteria represent the majority of the bacteria present in the nasal cavities. Additionally, there are opportunistic bacteria. Opportunistic bacteria retain the ability to act as commensals or pathogens. However, this depends on the integrity of the bacterial flora of the environment where they are found. For example, *Staphylococcus aureus* can become pathogenic following an alteration to the microbiome due to antibiotic therapies, pharmacological immunosuppression, or radiant therapies.

*Staphylococcus aureus* [23] colonizes approximately 30% of the human population asymptomatically in the nostrils, transiently or persistently. Therefore, it can be considered a human commensal [24].

## 4. Microbiota and Rhinosinusitis

CRS is a common and widespread inflammatory disease of the upper respiratory tract that significantly impacts the social aspect of life. It worsens the quality of life in everyday life and society, as well as negatively affecting public health costs.

The nasal microbiome related to CRS has been analyzed in different studies, which revealed a frequent presence of coagulase-negative *Staphylococcus, Pseudomonas*, and *Staphylococcus aureus* [25]. A recent study [26,27] analyzed the microbiome of patients with nasal polyps by comparing it to those of patients without nasal polyposis. The study established a prevalence of *Streptococcus, Haemophilus*, and *Fusobacterium* in patients without nasal polyps versus a predominance of *Staphylococcus, Alloiococcus,* and *Corynebacterium* in patients with nasal polyposis. 

In a recent study by De Boeck et al. [28], *Dolosigranulum pigrum* was clearly more associated with upper respiratory tract (URT) in healthy subjects, while *Corynebacterium tuberculostearicum, Haemophilus influenzae/H. aegyptius*, and *Staphylococcus* taxa were found to be more present in CRS patients. Understanding the mechanisms underlying the dysregulation of the nasal microbiome can be instrumental in both the clinical and post-operative evolution of patients with CRS. In patients with CRS, the microbiome has a reduced bacterial diversity, but a higher bacterial load.

Furthermore, less stable bacterial species replace more stable bacterial species (*Propionibacterium acnes*), favoring the colonization of potentially pathogenic bacteria [29,30]. The resulting dysbiosis could cause an alteration of the epithelial barrier, increasing its permeability to pathogens, followed by the release of inflammatory factors (cytokines and chemokines) with a consequent compromise of the immune system and chronic inflammation [31]. Moreover, some studies have demonstrated the presence of viruses and fungi in the mucosa of patients with CRS, which would contribute to increased adhesion of bacterial pathogens to the damaged mucosa [29,32,33,34,35]. Of great importance are the bacterial biofilms that are detected on the mucosa of patients with CRS. The development of a microbial biofilm is a complex process. Initially, sessile planktonic bacteria adhere to the mucosal surface and form microcolonies. Once they have taken root, the bacteria begin to proliferate and secrete an extracellular matrix composed of polysaccharides, nucleic acids, and proteins. This matrix protects the biofilm from harmful factors present in the environment. When bacterial density reaches a critical point, interbacterial cross-talk occurs, triggering a phenomenon known as “quorum sensing” or the “communication capacity of bacterial cells.” This phenomenon determines the biofilm phenotype that allows bacteria to communicate through small signal molecules to adapt to any change in the environment. The biofilm phenotype is morphologically characterized by the formation of microbial towers, composed of layers of live bacteria embedded within intermediate water channels. Bacteria in biofilms are more resistant to host defenses. The extracellular matrix that makes up most biofilm protects bacteria from antibodies, immune system phagocytosis, antibiotic penetration, and complement binding [36,37].

The biofilm may be pro-inflammatory through different mechanisms, including the release of planktonic organisms and the production of superantigens, which can cause ciliary dysfunction and the inhibition of mucociliary clearance [38].

The mucociliary clearance system represents a defense mechanism against inhaled particles. Therefore, its dysfunction favors the colonization of pathogenic bacteria and the establishment of inflammatory processes [39], contributing to the pathogenesis of CRS [30,40].

## 5. Probiotics

The WHO defines probiotics as products that contain living microorganisms that, when administered at the correct quantity, benefit the host’s health [8]. 

Probiotics should not be confused with prebiotics. Prebiotics are substances derived from foods that cannot be digested, whose beneficial effect on the host is their contribution to the growth, activity, or both of bacteria. 

The products containing prebiotics and probiotics are referred to as symbiotic [41]. The mechanism of action of probiotics has been described mainly in the gastrointestinal system, and includes several strategies through which they inhibit the action of pathogenic microorganisms. Probiotics may induce the inhibition of adhesion of pathogens to the mucous membranes, the stabilization of tight junctions in the epithelial layer with a reduction in the permeability of the mucosa, the competitive inhibition of pathogens, modulation of the immune system, and the production of various substances toxic to pathogenic microorganisms [42]. In a 2018 review, Martens et al. [43] described the possible mechanisms of action of probiotics in the respiratory tract, focusing on the positive effect of probiotics on the epithelial barrier and the immune system. They described the action of probiotics in restoring the epithelial barrier through the modulation of tight junctions and adherence junctions and their role in modulating the host’s immune response through their interaction with dendritic cells. This promotes regulatory T-cells (Tregs) and downregulates T-helper 1 and T-helper 2. In studies evaluating potential probiotics, the ability to adhere to the nasal epithelium, that is, the ability to survive in aerobic conditions and at low temperatures, must be considered [44]. These conditions are necessary for the probiotics to compete with opportunistic bacteria such as *Staphylococcus aureus*.

## 6. Clinical Studies

### 6.1. Clinical Studies That Used Oral Administration of Probiotics

Habermann et al., in 2002 [45], conducted a double-blind, placebo-controlled, multicenter study on the efficacy of human *Enterococcus faecalis* (Symbioflor^®^1) in reducing the frequency of exacerbations of CRS in a sample of 157 patients. Half of the patients were treated with the oral administration of drops containing the probiotic for six months. The other half of the patients were treated with placebo also for six months. After eight months of follow-up, there were approximately half of the exacerbations in patients treated with human *Enterococcus faecalis* compared to patients treated with placebo. 

In another prospective, randomized, double-blind, placebo-controlled trial, Mukerji et al. in 2009 [46] used the oral administration of a probiotic strain of *Lactobacillus rhamnosus R0011* (500 million active cells in tablets, twice a day) for four weeks in a group of 38 patients with CRS; 39 CRS patients represented the placebo-treated control group. The authors used the SNOT-20 quality of life test to assess the effectiveness of the treatment. After four weeks, patients treated with the *Lactobacillus* probiotic reported a better quality of life than the control group. However, this benefit was not confirmed over time, as after eight weeks, there were no significant differences in the responses to the quality-of-life test between the two groups (Table 2).

### 6.2. Clinical Studies That Used Local Administration of Probiotics

Martensson et al. in 2017 [47], in a randomized, double-blinded, crossover, and sham-controlled study, evaluated the effects of administration through a nasal spray of Honeybee lactic acid bacteria (LAB). Honeybee LAB, consisting of various *Lactobacilli* and *Bifidus bacteria*, was administered to 20 patients with CRSsNP for two weeks. The efficacy of the treatment was assessed by considering the trend of the symptoms through the use of the SNOT-22 questionnaire. The impact of the treatment on the microbiome and inflammation products (IL-6, IL-8, and TNF-9) was evaluated using the nasal wash fluid. The treatment proved to be well tolerated. However, it was not effective in reducing symptoms, nor did it affect the microbiota composition. There was no change in the inflammation processes. 

Endam et al. [48] conducted a prospective open-label pilot trial of the safety and feasibility study. The authors aimed to verify if topical administration of *Lactococcus lactis W 136* for 14 days to the nasal and sinus cavities would be safe for patients with CRS refractory to medical and surgical treatment. The evaluation of symptoms was performed with the SNOT-22 test. 

Simultaneously, an endoscopy nasal was carried out to evaluate the conditions of the mucosa nasal and the UPSIT-40 test to detect the olfactory function. The treatment turned out to be well tolerated by all 24 patients, and was found to improve symptoms that remained 14 days after the end of the course of treatment with the probiotic, while the sense of smell remained stable (Table 3).

## 7. In Vivo and In Vitro Experimental Studies

In 2016, Schwartz et al. [49] evaluated the capacity of the two Gram-positive probiotics strains of *Lactococcus lactis* to stimulate the production of IL-10 and TNF on preparations of peripheral blood monocytes (PBMC). Furthermore, the authors assessed the safety of applying *Lactococcus lactis* on the mucosal cells of the paranasal sinuses of patients with and without rhinosinusitis. These in vitro studies have supported the safety and immunomodulatory capacities of *Lactococcus lactis* for intranasal use. The cultures of cells of the mucosa of the paranasal sinuses of patients with and without CRS showed no evidence of toxicity when exposed to the supernatant of this strain. Conversely, the preparations of peripheral blood monocytes showed the induction of IL-10 and TNF without evidence of toxicity or excessive Th1-type inflammation. The authors concluded by stating that topical nasal therapy could represent a new therapeutic strategy for patients with CRS. In an in vitro study, Cho et al. [50], in 2020, assessed the growth of six strains of *Pseudomonas aeruginosa* derived from patients with CRS (three patients with cystic fibrosis, and one patient with ciliary dyskinesia)—the first strain of *Pseudomonas aeruginosa* from the laboratory. These strains were co-cultured with *Lactococcus lactis* (obtained from commercial probiotic nasal washes) in the presence of mucin.

Many *Pseudomonas aeruginosa* strains were grown without *Lactococcus lactis* (control cases). No influence on the growth of *Pseudomonas aeruginosa* colonies was observed in cultures where *Lactococcus lactis* was present. The growth inhibition of *Pseudomonas aeruginosa* was observed only in one culture found to be contaminated with *Stenotrophomonas* *maltophilia*. The authors concluded that nasal lavage with probiotics (*Lactococcus lactis*) may not be helpful for all patients. Therefore, further experiments are needed to evaluate the interactions between *Pseudomonas aeruginosa* and *Lactococcus lactis*. 

In 2012, Abreu et al. [51] conducted a study to determine whether *Corynebacterium tuberculostearicum* exhibited pathogenic potential and whether this could be affected by the resident microbiota. They developed a mouse model of sinus infection using goblet cell hyperplasia and mucin hypersecretion as markers of pathology.

Nasal inoculation of large numbers of *Corynebacterium tuberculostearicum* in the presence of a complete (healthy) sinus microbiota resulted in an increase in the number of mucin-secreting goblet cells. Animals treated with both an antibiotic (to reduce the bacterial load in the microbiota) and *Corynebacterium tuberculostearicum* showed profound goblet cell hyperplasia. To demonstrate that goblet cell hyperplasia and mucin hypersecretion were explicitly induced by *Corynebacterium tuberculostearicum*, they repeated the experiment by adding a group of antibiotic-treated murine models before nasal inoculation of *Lactobacillus sakei*, which is present in abundance in healthy mucosal samples and significantly reduced in CRS patients. Sinus mucosal histology demonstrated that the group treated with antibiotics and inoculated with *Corynebacterium tuberculostearicum* showed significant increases in goblet cell hyperplasia and mucin hypersecretion. However, mice that received identical numbers of *Lactobacillus sakei* demonstrated epithelial physiology comparable to that of control animals (no significant difference in the number of goblet cells), thus confirming that the observed sinus histopathology was explicitly due to *Corynebacterium tuberculostearicum*. Furthermore, species such as *Lactobacillus sakei* protect the epithelium of the rhino-sinus mucosa through competitive inhibition of *Corynebacterium tuberculostearicum*. In 2014, Cleland et al. [52] investigated the probiotic properties of *Staphylococcus epidermidis* against *Staphylococcus aureus* in murine sinusitis models. 

They demonstrated that *Staphylococcus epidermidis* exerts a probiotic effect by producing a serine protease that inhibits biofilm production and *Staphylococcus aureus* colonization. Even in this case, the hypertrophy of muciparous cells and their hypersecretion as well as the characteristics of the CRS were considered markers of inflammation. This study showed that *Staphylococcus epidermidis* can be a potential probiotic, having induced, in a murine sinusitis model, reduced counts of goblet cells in a group of mice co-inoculated with *Staphylococcus epidermidis* + *Staphylococcus aureus* compared to those who receive only *Staphylococcus aureus* (Table 4).

## 8. Conclusions

In the literature, there are still few and conflicting studies on the efficacy of probiotics in acute inflammatory diseases of the upper airways and, in particular, in CRS. The studies available to date are also based on small sample sizes. Only three of the studies described above, of which two are in vivo and one in vitro, described a beneficial effect of treatment with probiotics on CRS. Animal studies highlight the ability of some probiotics to reduce the inflammatory phenomena of CRS on the mucosa. To the best of our knowledge, no other data in the literature can illustrate the long-term effect of probiotics on CRS. Further efforts will undoubtedly have to be made to evaluate the potential of probiotics on CRS. Indeed, the study of the microbiota of affected patients and bacterial biofilm will have to continue. 

## Figures and Tables

**Table 1:** Commensal bacteria, represented genera in the different sites of the nose.

Sites of the Nose	Commensal Bacteria
Vestibules	*Corynebacterium, Propionibavterium, Staphylococcus*
Nasal cavities	*Staphylococcus, Corynebacterium, Dolosigranulum*
Nasopharynx	*Moraxella, Streptococcus, Fusobacterium, Haemophilus*

**Table 2:** Clinical studies on the treatment of CRS with probiotics—Oral administration.

Author	Type of Study	Probiotic	N. Patients	Results
Habermann et al., 2002	Multicenter, randomized, double blind, placebo controlled trial	*Enterococcus faecalis*	157	Reduction of CRS flare-ups
Mukerji et al., 2009	prospective, randomized, double-blind, placebo-controlled trial	*Lactobacillus rhamnosus*	77	Transient improvement in the quality of life

**Table 3:** Clinical studies on the treatment of CRS with probiotics—Local administration.

Author	Type of Study	Probiotic	N. Patients	Results
Martensson et al., 2017	randomized, double-blinded, crossover, and sham-controlled trial	*Honeybee lactic acid bacteria*	20	Not effective
Endam et al., 2020	Prospective open-label pilot trial of safety and feasibility	*Lactococcus lactis*	24	Transient improvement in CRS symptoms

**Table 4:** In vitro and in vivo experimental studies on probiotics activity.

Author	Type of Study	Probiotic	Conclusions
Schwartz et al., 2016	In vitro study	*Lactococcus lactis*	Absence of cellular toxicity, induction of IL-10 and TNF
Cho et al., 2020	In vitro study	*Lactococcus lactis*	Lactis nasal washes may not be helpful for all CRS patients
Abreu et al., 2012	In vivo study (mouse)	*Lactobacillus sakei*	Treatment with *L.sakei* could counteract the action of *C. tuberculostearicum*
Cleland et al., 2014	In vivo study (mouse)	*Staphylococcus epidermidis*	*S. epidermidis* inhibits the colonization of *S. aureus*
